# Digital Care in Epilepsy: A Conceptual Framework for Technological Therapies

**DOI:** 10.3389/fneur.2018.00099

**Published:** 2018-03-02

**Authors:** Rupert Page, Rohit Shankar, Brendan N. McLean, Jane Hanna, Craig Newman

**Affiliations:** ^1^Dorset Epilepsy Service, Poole Hospital NHS Foundation Trust, Poole, United Kingdom; ^2^Cornwall Partnership NHS Foundation Trust, Truro, United Kingdom; ^3^Exeter Medical School, Knowledge Spa, Royal Cornwall Hospital, Truro, United Kingdom; ^4^Royal Cornwall Hospital, Truro, United Kingdom; ^5^SUDEP Action, Wantage, United Kingdom; ^6^Plymouth University Peninsula Schools of Medicine and Dentistry, Plymouth, United Kingdom

**Keywords:** epilepsy technology, automated epilepsy risk monitoring, electronic health platforms, mobile apps, Epilepsy Self-Monitor, self-empowerment, co-production of health records

## Abstract

Epilepsy is associated with a significant increase in morbidity and mortality. The likelihood is significantly greater for those patients with specific risk factors. Identifying those at greatest risk of injury and providing expert management from the earliest opportunity is made more challenging by the circumstances in which many such patients present. Despite increasing recognition of the importance of earlier identification of those at risk, there is little or no improvement in outcomes over more than 30 years. Despite ever increasing sophistication of drug development and delivery, there has been no meaningful improvement in 1-year seizure freedom rates over this time. However, in the last few years, there has been an increase in patient-triggered interventions based on automated monitoring of indicators and risk factors facilitated by technological advances. The opportunities such approaches provide will only be realized if accompanied by current working practice changes. Replacing traditional follow-up appointments at arbitrary intervals with dynamic interventions, remotely and at the point and place of need provides a better chance of a substantial reduction in seizures for people with epilepsy. Properly implemented, electronic platforms can offer new opportunities to provide expert advice and management from first presentation thus improving outcomes. This perspective paper provides and proposes an informed critical opinion built on current evidence base of an outline techno-therapeutic approach to harnesses these technologies. This conceptual framework is generic, rather than tied to a specific product or solution, and the same generalized approach could be beneficially applied to other long-term conditions.

## Introduction

### Clinical Challenges

Epileptic seizures are a manifestation of bulk electrochemical discharges within the brain and symptomatic of a wide range of different possible neurological and other physical disorders. Consequently, for presentations with paroxysmal neurological symptoms, particularly involving alteration of consciousness, epilepsy is a diagnostic consideration. The potential manifestations of these discharges are protean and can be diagnostically difficult even for epilepsy specialists. The core of accurate diagnosis of the disorder is based on history. Contemporaneous witness accounts are crucial, but the availability and accurate recall of the latter decays significantly over time. In time-pressured environments such as primary care and the emergency settings where the first presentation occurs, diagnostic accuracy may be little better than chance (1–3). Studies from the UK and elsewhere in Europe have consistently revealed that people with epilepsy (PwE) experience significant difficulties in accessing specialists in an emergency setting (4).

**Figure 1:**
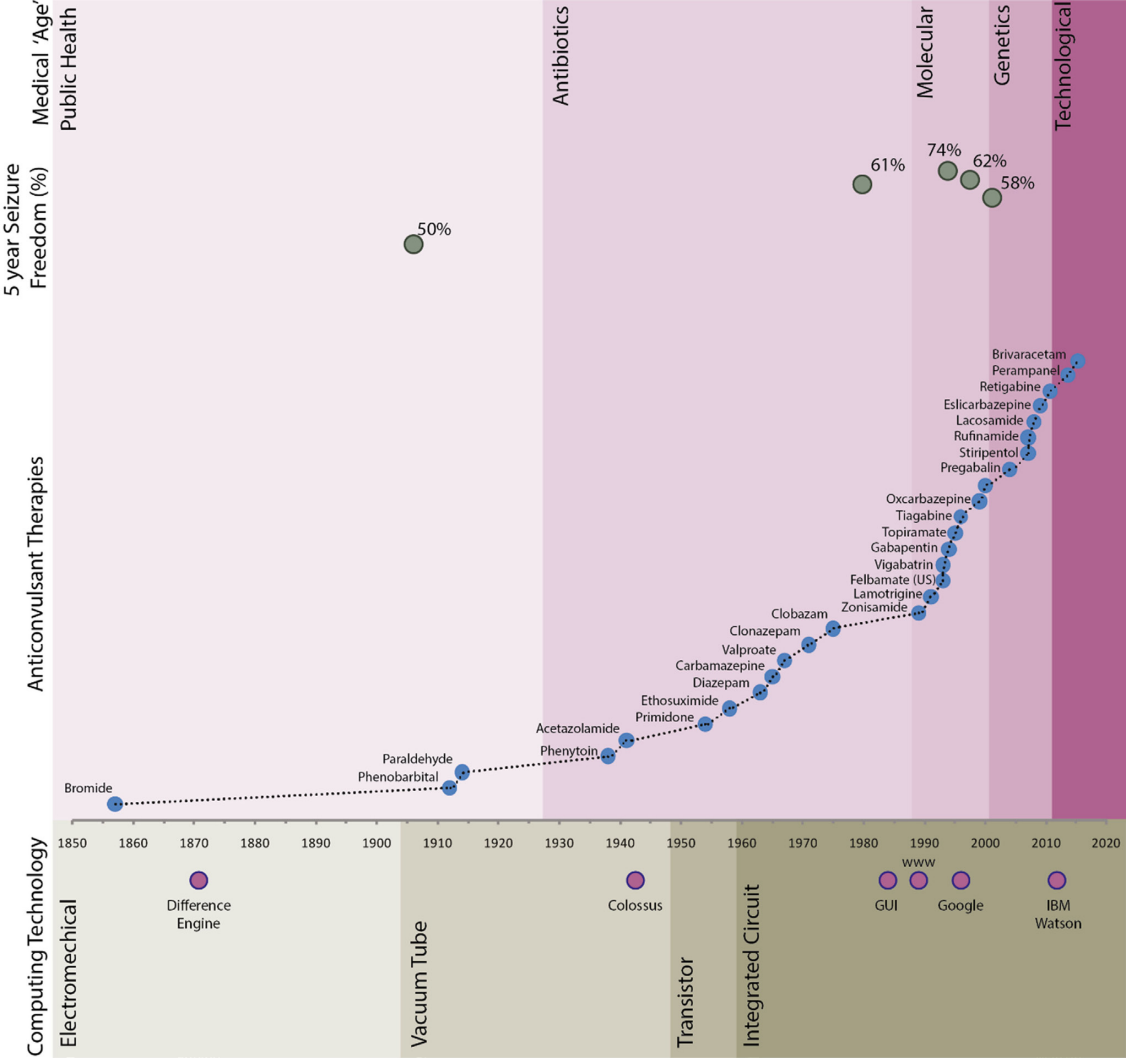
Long-term seizure freedom, anticonvulant therapy agent availability, and computing timeline. Seizure freedom data sources referenced in text. Difference engine—Chales Babbage; Colossus—Bletchley Park; graphical user interface (GUI)—developed from Xerox PARC in around 1981 and popularly implemented by companies including Apple and Microsoft; Google—founded in 1998; IBM Watson—natural language medical artificial intelligence system developed by IBM.

Epilepsy has been recognized since antiquity, but the first broadly effective treatment was identified less than 200 years ago. Over 20 chemicals with anticonvulsant properties have made been used widely, with more than half of these available in the last 20 years (Figure 1). Despite their increasing pharmacological specificity, these agents have not led to a significant increase in the proportion of patients who are seizure free (5). Delays in referral for epilepsy surgery or other specialized approaches for those with drug-refractory epilepsy are a common finding (6, 7).

**Figure 2:**
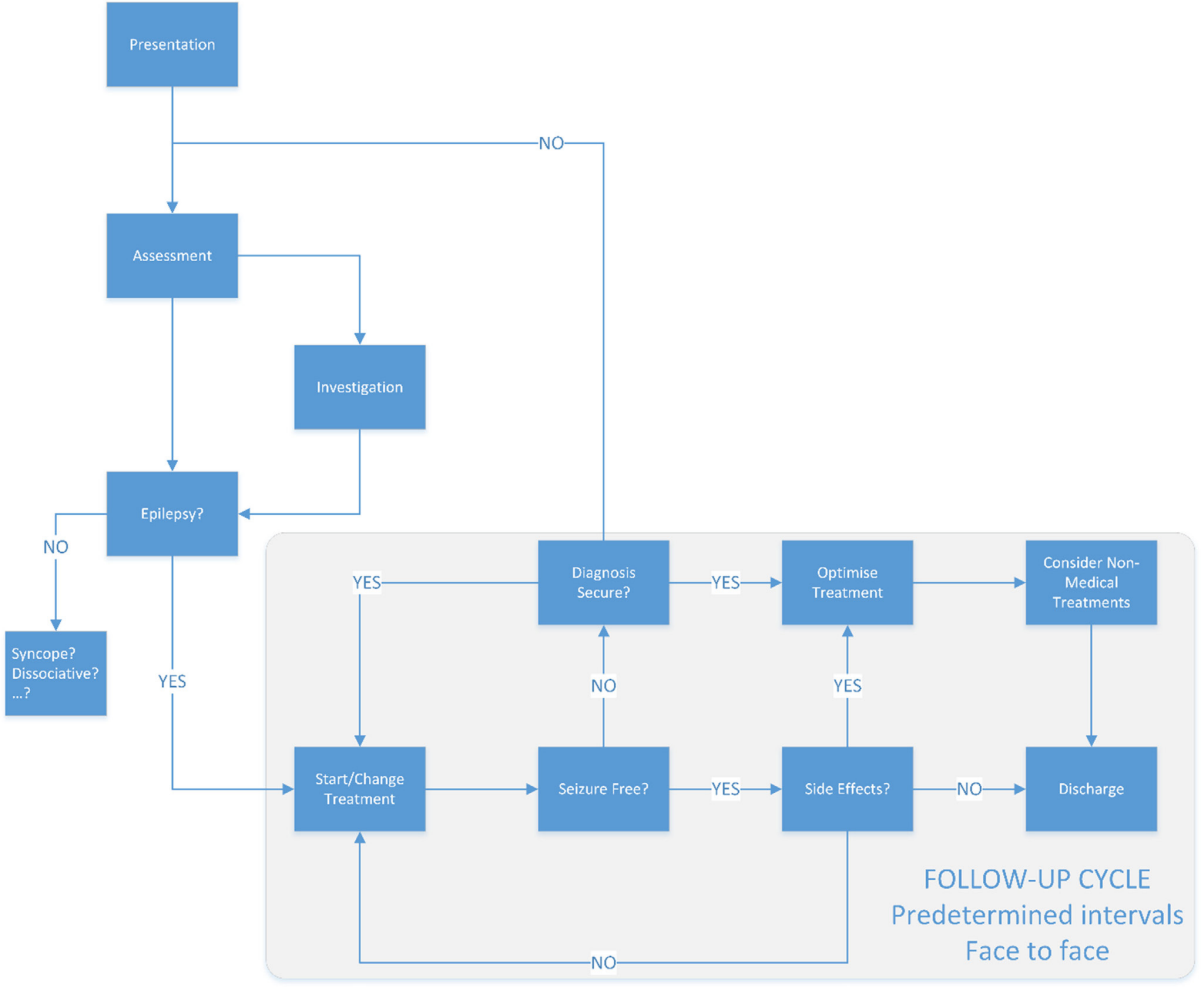
The traditional model of epilepsy care. Typically once a patient has a diagnosis of epilepsy, the follow-up will be often at in person at predetermined intervals—typically 3 or more months apart.

Much of how outpatient-based neurological care is delivered today would be familiar to John Hughlings Jackson (1835–1911) (Figure 2). Over the last 30 years, technology has made rapid advances. The confluence of developments in material and computer science has contributed to a plethora of devices and applications that now seem mundane. Medicine in general has been slow to adapt to these changes, generally developing modish and superficial applications that ape aspects of existing clinical care provision (8, 9). For epilepsy, the focus has primarily been on real-time seizure detection, electronic versions of paper forms or diaries (10). Implantable or wearable stimulators are currently the limit of therapeutic technological intervention. These approaches, while laudable in aim, often fail to integrate into clinical care pathways resulting in increased workload for already busy clinicians and do not deliver on their promise. Comprehensive reviews of these technologies are available (11).

The challenges of recruiting, training, and retaining specialist clinical staff to support patients with long-term conditions such as epilepsy is difficult and likely to become more so (12). Around 70% of the cost for any Acute Trust in the UK is staff, thus leveraging the existing workforce to support patients in the community is essential to maintain service provision.

### Technical Challenges

Changing existing working practices is difficult, even when the reasons to do so are clear (13). Medicine is not immune to this problem. Attempts to introduce electronic health records (EHRs) have been bedeviled by difficulties (14–16). An increasing body of evidence has shown that poorly thought out, and implemented systems and clinical user interfaces have led to time being lost entering data into the EHR, potentially with a detrimental effect on the interaction between patient and clinician (17, 18). Patient contributed data, such as seizure or symptom diaries, are often in paper format. If it is in electronic form, it usually cannot be easily integrated into the clinical record.

While clinicians may not be able to change their EHR, patients are free to change between different applications and technology according to their needs and personal preferences. Surveys from the US suggests that over 20% of mobile smartphone apps are used only once in 6 months (19). By comparison, the neurology outpatient clinic “did not attend” rate is typically around 7% (20). If patients are to be safely engaged and retained in clinical follow-up using a technological solution, then this must be easy to use and of value to them. From a technical perspective, it needs to meet national security requirements for data both at rest and in transit. In addition, the patient contributed data must be able to be correctly registered against clinical EHRs from different vendors when the patient changes their care provider, as well as being able to share this with different teams within the care economy.

### Patient Challenges

Technology has had a pervasive influence on the modern world, from the Internet to autonomous vehicles. There is an increasing acceptance of “electronic first” approaches to communication and interaction in the consumer sphere, although it is only lately being accepted in the medical realm. The widespread availability of smartphone technology and focused user experience (UX) design has undoubtedly made for a more seamless experience for people of all ages. Despite the widespread availability of technology, there remains a significant minority who prefer to avoid technological solutions. The size of this latter group is likely to diminish over time due to increasing familiarity and demographics. The provision of Wi-Fi or broadband connectivity, particularly in remote or rural areas is often poor. In some areas of the UK, a cellular signal remains unavailable. An electronic rather than paper-based system also poses many challenges to the traditional dynamics of a patient–clinician communication, not least the loss of eye-contact that frequently results. Unless managed sensitively, this can adversely impair the ability of the practitioner to obtain clinical information critical to diagnosis and management.

### Opportunities

**Figure 3:**
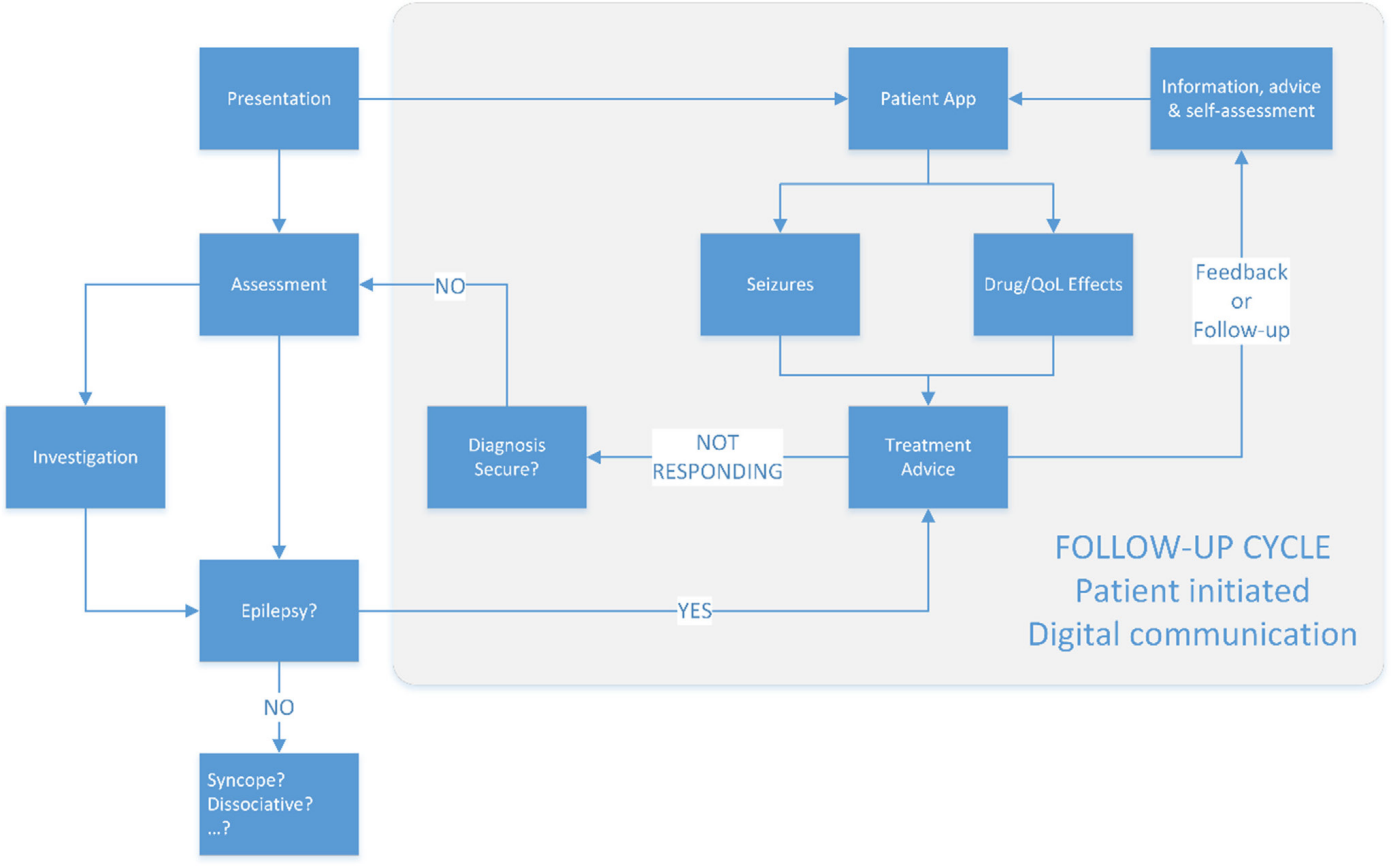
The proposed new model of epilepsy care. Follow-up is patient triggered, supported by design to ensure that alerts to the clinical team are notified in proportion to their severity on an evidenced based approach.

A patient co-authored record has the potential to reduce clinical data entry requirements while increasing the relevance of that record (Figure 3). Paired with instant communication and alert thresholding, patients with long-term conditions such as epilepsy can be safely managed on a patient-triggered follow-up basis. The real-time nature of the communication with specialists can reduce the risk of preventable harms to patients, including sudden unexpected death in epilepsy (SUDEP) (21, 22).

The addition of dynamic and informed patient consent to this coauthored record opens the possibility of real-world post-marketing surveillance studies at a fraction of the cost of traditional pharmaceutical trials (23).

## Concept

To take advantage of the opportunities technology may offer, it is necessary to reconsider the traditional approach to managing patients presenting with possible epilepsy. Current best practice in the UK recommends patients presenting with a possible first seizure to be referred to a specialist and to be seen “as soon as possible” (24). Any investigations required including imaging are expected to be undertaken “soon” after they are requested. A pathway to take advantage of the new technology begins at the first presentation with a possible seizure and uses commonly available platforms to facilitate rapid assessment, risk stratification, and communication.

The conceptual framework offered is intended to be generic in outline, rather than focused on a specific device or platform. It describes a hypothetical technology-enabled “scaffold” on which a more modern and timely epilepsy service might be built. The current rate of technological development means that present offerings may rapidly be overtaken by other approaches. The aim of this paper therefore is to suggest a standardized approach that can be employed across a shifting framework of devices and platforms, to the consistent benefit of PwE. Where relevant, it uses the direct experience of the authors to evidence the benefits that may be derived.

### At First Presentation

A majority of first seizure presentations in the UK will be seen or referred to hospital. Following clinical assessment, patients with possible first seizure can be offered a suite of downloadable resources for seizures and epilepsy that reflect both national best practice and local guidelines with signposting. These resources should include an electronic symptom/seizure diary application, which is registered to the local EHR for the Epilepsy Team. The data collected at the time of presentation should include information which can be used to stratify patients for seizure/SUDEP risk, based on published evidence and validated tools (22, 25). This information should be included in the electronic referral, which is notified to the First Seizure Clinic and Epilepsy Team. Outpatient investigation and appointment details are sent to the patient electronically *via* the smartphone diary application. Relevant information recorded in the patient smartphone application is communicated to the care team, using the updated risk stratification. This information may include manually recorded possible seizures, witness accounts, and video as well as data from wearable devices. Investigations, advice, and management are adjusted based on the up-to-date information, with the timing of first clinical review adjusted according to the live recurrence risk.

Work done by the Epilepsy Self-Monitor (EpSMon) collaboration has shown that patients can be engaged with a smartphone application on a regular basis and that they can use it to help reduce their risk of injury and death through uncontrolled epileptic seizures.

### Clinical Notification and Messaging

Clinically relevant updates entered *via* registered patient smartphone application(s) need to be notified to the Epilepsy Team. These updates require automatic grading using evidence-based markers of seizure severity morbidity. Risk stratifying the alerts handling in the clinical application, combined with the agreed clinical goals of treatment, prevents alert notification fatigue for the Epilepsy Team and focuses attention on the “at need” population. Lower risk updates are summated on a weekly, monthly, or 3-monthly basis agreed with the patient and care team, with an established escalation process for unexpected worsening. These lower risk updates are commented on by the Epilepsy Team, and this feedback is passed to the patient or care team through the system. The ongoing real-time feedback loop is required to maintain patient engagement and update treatment plans to optimize care.

The Epilepsy Care Alliance has demonstrated a smartphone application can be used to leverage limited clinical resources to provide real-time advice to patients with epilepsy. In doing so, the rate of hospital admission for patients known to have epilepsy has fallen by over 30% (Page, unpublished data).

### Coauthored Record

Where appropriate, wearable devices may be used to help supplement patient provided data. This may be in terms of improving seizure recording, for instance, in terms of nocturnal seizures, or by logging lifestyle data around activities such as exercise and sleep. Patients need to be able to confirm or deny putative seizures recorded by a wearable device as these currently will not reliably record all seizures and are prone to false positives. A daily summary of the relevant life-logging data provided should be registered against the clinical record, where appropriate patient consent is obtained.

Semiautomated clinical assessment of the data obtained would permit meaningful personal insights into aspects such as possible seizure triggers and medication side effects. Such approaches may facilitate statistically valid correlations in individuals if there is a sufficiently large data set obtained. Patients would need to assess their seizure severity and risk of SUDEP using a clinically validated approach, such as EpSMon (10, 26). This provides them with details of modifiable and non-modifiable risks. The assessment should be updated at regular intervals to provide an insight into risks that they can modify.

### Ongoing Care

Engaging patients with a technological approach to any long-term condition requires awareness of behavioral trends for the population to minimize drop-out of “at need” patient groups. This may include techniques such as online training videos and refresher modules. It requires a process for reaching out to patients who may become disengaged from the electronic process or find it too cognitively taxing to commit to. In addition, an ability to update the “techno-therapy” to both take advantage of new developments in medical, computing, and material science, as well as a constantly shifting series of operating systems and physical platforms is essential to maintain clinical utility and patient usability.

For epilepsy, successful implementation of the techno-therapeutic approach described should allow patients to be assessed once in a traditional face-to-face clinic with subsequent “follow-up appointments” being completed using asynchronous text or data-based approaches or using technologies such as Skype, apart from a small subset who may need face-to-face review. With the development of augmented/blended reality approaches even this requirement may shrink.

The transition from traditional follow-up to a patient-triggered follow-up process will require some reconfiguration of clinical services. This would be expected to include more time focused on surveillance of patient contributed data using automation, data analysis tools, and structured notifications, with relatively less time spent in face-to-face review.

### Evidence Base

The EpSMon (22) is a digital self-assessment smartphone app which comprehensive review of technological devices for epilepsy has been published elsewhere (11, 27). This conceptual framework has been influenced by the clinical experience of the authors in developing approaches to help advance technology-enabled care. A summary of this is outlined below.

The EpSMon (28) is a digital self-assessment smartphone app, which provides PwE access to a patient facing version of the clinical epilepsy risk checklist (22). Users are prompted to assess their risk status relative to the current evidence on risk of SUDEP with resultant education and suggestions to seek appropriate levels of clinical contact when appropriate. EpSMon has been downloaded by 4,000 users. Recognition that EpSMon would likely best fit within existing NHS care has been supported by slower than expected take-up, technology peer review (11, 27) (NIHR) and commissioning of the project into NHS England’s innovation accelerator program (29). Additional learning prompts expansion of the technology to include a website version (for increased accessibility), increased epilepsy management features (medication reminder and seizure log), and increased interoperability with patient flagging or data management systems.

The Epilepsy Care Alliance has developed an electronic system for managing PwE, using patient smartphone application and wearable technology deployed to a small pilot group of PwE. This is combined with team-based messaging that notifies of any patients admitted to Poole Hospital known to the Dorset Epilepsy Service. This has been running since September 2016 and is being actively updated. There was a 30% reduction in admissions to Poole hospital for PwE known to the epilepsy service. In addition, in those patients who were using the electronic app, there was a reduction in the median interval to medication adjustment of over 3 weeks compared with before introduction of the technology. The impact on quality of life and seizure freedom attainment rates in this group are currently the subject of ongoing analysis.

## Discussion

Technological “solutions” for patients typically focus on a specific disease or condition. There is frequently a varying degree of clinical certainty as to the diagnosis. This uncertainty is typically most pronounced at the initial presentation, due to a combination of missing/misleading information and lack of specialist input. This is particularly true for epilepsy, a condition with is characterized by both altered awareness of the patient and the unpredictability of seizures. To ensure that patients are not placed at avoidable risk due to clinical uncertainty at the outset, “prescribing” an application suited for presentations of altered awareness including high risk conditions such as epilepsy is prudent. The use of open application programming interfaces enables data that are already entered to be moved to other disease-specific applications as diagnostic certainty increases. Such an open standard for data facilitates easier sharing of information between different applications. It would also help foster an ecosystem of patient applications, which can evolve to keep pace with the needs of patients.

From a public health perspective, it is often difficult to determine the real-world frequency of disorders and their eventual outcomes. Case selection is fraught with bias depending on the source of the study. Long-term follow-up can be challenging and is often resource and cost prohibitive. Most long-term conditions, including many causes of epilepsy, are believed to persist for the remainder of life after diagnosis. The “gold-standard” for epilepsy is typically 1-year seizure freedom, with the risk of seizure recurrence thought to progressively drop in subsequent years. Most patients would not be followed-up beyond 2 or 3 years of seizure freedom. Data on late seizure recurrence and life-long seizure freedom as well as long-term impact of many anticonvulsant medications are lacking. Changing the way in which the health-care system interacts with people with long-term conditions from their first presentation through the rest of their life, based on their needs, provides an opportunity to assess the true impact of lifestyle, disease, and clinical interventions on quality of life. One would expect that this would provide new evidence and insights that may radically change future care and advice.

The transition to a “digital first” architecture for clinicians and patients requires technological transparency. This is the means of data collection facilitates the clinical assessment rather than dominating it. Providing a smartphone-based application for the patient contributed part of the record is an approach that fits the consumer trend of technology uptake and use, while reducing costs to the health-care economy. In view of the rapidly evolving nature of computing, machine learning, and materials science, opportunities for improvements in health care are likely to increase faster through such approaches than advances in traditional medical research and drug development. The shifting population demographic and focus on UX would be expected to progressively reduce the “digitally disenfranchised.” The current orthodox method of long-term care provision will undoubtedly change over the coming years. While these changes need to be inclusive and sustainable and clinically led they must be alert to the possibilities that technological development can enable.

## Ethics Statement

We confirm that we have read the journal’s position on issues involved in ethical publication and affirm that this report is consistent with those guidelines. No ethical approval was needed.

## Author Contributions

RP and RS wrote the initial draft. It was edited and developed further by BM, JH, and CN. All the authors have contributed to the development, concept, writing, editing, and delivery of the paper.

## Conflict of Interest Statement

RP is part of the Epilepsy Care Alliance (a collaboration of Poole NHS Foundation Trust, Kent University, Graphnet, and Shearwater) with no private profit. RP has received research funding from Innovate UK and the Wessex Academic Health Science Network. RP has received personal and educational funding from UCB, Eisai, GSK, and Orion. RS and JH are the main stakeholders of the “SUDEP and Seizure Safety Checklist.” RS, JH, BM, and CN are developers and key stakeholders of EpSMon. RS has received institutional and research support and personal fees from LivaNova, UCB, Eisai, Special Products, Bial, and Desitin outside the submitted work. BM has received research support and personal fees from Eisai, UCB, and Desitin outside the submitted work. CN receives research support from the NIHR National Innovation Accelerator program. The reviewer JZ and handling Editor declared their shared affiliation.

## Abbreviations

DNA, did not attend; EHR, electronic health record; EpSMon, Epilepsy Self-Monitor; PwE, people with epilepsy; SUDEP, sudden unexpected death in epilepsy; UX, user experience.
